# Critical care beyond organ support: the importance of geriatric rehabilitation

**DOI:** 10.1186/s13613-024-01306-1

**Published:** 2024-05-10

**Authors:** Jeremy M. Jacobs, Ana Rahamim, Michael Beil, Bertrand Guidet, Helene Vallet, Hans Flaatten, Susannah K. Leaver, Dylan de Lange, Wojciech Szczeklik, Christian Jung, Sigal Sviri

**Affiliations:** 1 Department of Geriatric Rehabilitation and the Center for Palliative Care. Hadassah Medical Center and Faculty of Medicine, Hebrew University of Jerusalem, Jerusalem, Israel; 2 Geriatric Unit, Hadassah Medical Center and Faculty of Medicine, Hebrew University of Jerusalem, Jerusalem, Israel; 3 Department of Medical Intensive Care, Hadassah Medical Center and Faculty of Medicine, Hebrew University of Jerusalem, Jerusalem, Israel; 4 Assistance Publique - Hôpitaux de Paris, Hôpital Saint-Antoine, Service de Réanimation Médicale, Paris, France; 5 Sorbonne Universités, UPMC Univ Paris 06, UMR_S 1136, Institut Pierre Louis d’Epidémiologie et de Santé Publique, Paris, France; 6 Department of Geriatrics, Centre d’immunologie et de Maladies Infectieuses (CIMI), Institut National de la Santé et de la Recherche Médicale (INSERM), UMRS 1135, Saint Antoine, Assistance Publique Hôpitaux de Paris,, Sorbonne Université, Paris, France; 7 Department of Clinical Medicine, University of Bergen, Bergen, Norway; 8 Department of Research and Development, Haukeland University Hospital, Bergen, Norway; 9 General Intensive Care, Department of Critical Care Medicine, St George’s NHS Foundation Trust, London, UK; 10 Department of Intensive Care Medicine, University Medical Center, University Utrecht, Utrecht, The Netherlands; 11 Center for Intensive Care and Perioperative Medicine, Jagiellonian University Medical College, Kraków, Poland; 12 Department of Cardiology, Pulmonology and Vascular Medicine, Faculty of Medicine, Heinrich-Heine-University, Moorenstraße 5, 40225 Düsseldorf, Germany

**Keywords:** Critical care, Old patients, Geriatric rehabilitation, Comprehensive geriatric assessment, Frailty, Post-ICU syndrome

## Abstract

Very old critically ill patients pose a growing challenge for intensive care. Critical illness and the burden of treatment in the intensive care unit (ICU) can lead to a long-lasting decline of functional and cognitive abilities, especially in very old patients. Multi-complexity and increased vulnerability to stress in these patients may lead to new and worsening disabilities, requiring careful assessment, prevention and rehabilitation. The potential for rehabilitation, which is crucial for optimal functional outcomes, requires a systematic, multi-disciplinary approach and careful long-term planning during and following ICU care. We describe this process and provide recommendations and checklists for comprehensive and timely assessments in the context of transitioning patients from ICU to post-ICU and acute hospital care, and review the barriers to the provision of good functional outcomes.

**Supplementary Information:**

The online version contains supplementary material available at 10.1186/s13613-024-01306-1.

## Introduction

Very old patients (aged 80+) are the fastest growing patient population in intensive care in many countries [1]. Critical illness and the burden of treatment in the intensive care unit (ICU) can lead to a long-lasting decline of functional and cognitive abilities, especially in the very old, frail patient with reduced resilience to stress [2, 3]. The restoration of functional integrity and the associated improvement in overall quality of life are considered to be both the goal and the main patient-centred outcome measure among this age cohort. Thus, rehabilitation that supports this process is a key component of managing critical conditions in very old individuals. Evidence suggests that early mobilization and rehabilitation improves patient-centred outcome measures [4–6]. The process of rehabilitation should start in the ICU and continues far beyond discharge. It requires coordination between intensive care and geriatric medicine at both the in-patient and out-patient settings to employ the full armamentarium of the latter in the most effective way [7, 8]. This narrative review will focus on rehabilitation for very old ICU patients and will discuss steps required in the ICU to prepare these geriatric patients for rehabilitation following discharge.

## Geriatric rehabilitation

Very old critically ill patients typically present the greatest clinical and rehabilitation challenges due to complex multi-morbidity and a substantially increased vulnerability to stress [9]. Rehabilitation delivers a range of complementary tailored interventions to attain the goal of optimal physical and cognitive function with minimal disability among people with impairments [10].

**Fig. 1:**
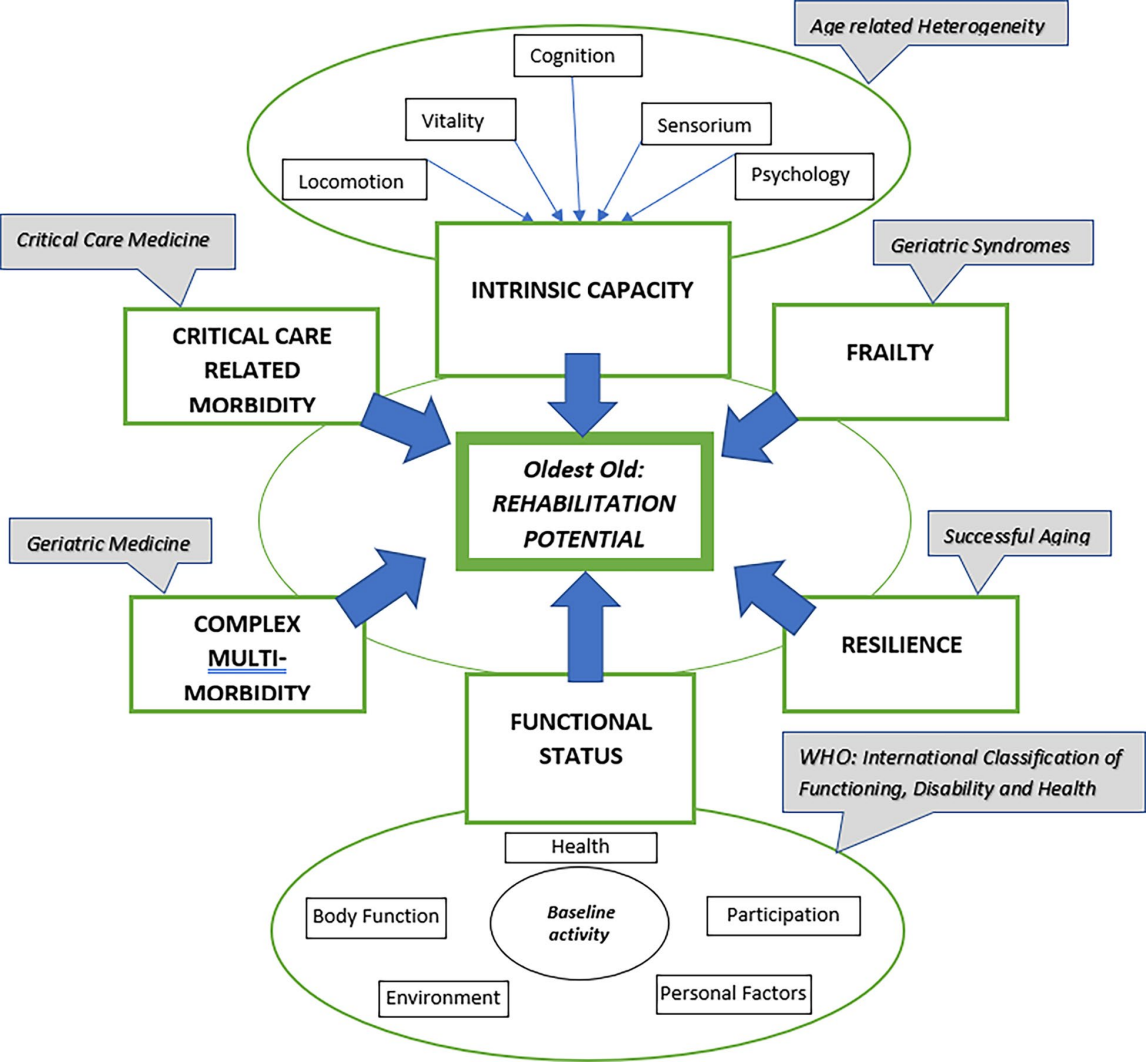
A proposed conceptual framework for rehabilitation potential of very old patients in the ICU. The proposed framework recognizes the multiple factors which influence rehabilitation potential of very old patients in the ICU. In addition to specific considerations of both critical and geriatric medical care, the individual's baseline functional status and level of activity is recognized to be an important determinant of subsequent rehabilitation potential. The emerging concept of intrinsic capacity, reflecting vital domains which display a wide heterogeneity among older people, is also introduced into the proposed framework, in addition to the modulating factors of frailty and resilience

In the last 20 years, the World Health Organization has adopted the International Classification of Functioning, Disability and Health (ICF), which represents a conceptual shift from the classical model of "disease-impairment-disability-handicap" [11]. The ICF model emphasizes a multiplicity of interacting synergistic factors, which lead towards the final common endpoint of disability (Fig. 1). Placed within the ICF concept, rehabilitation must address the five interacting domains of “health conditions”, “body functions and structure”, “participation”, “personal factors” and “environmental barriers”. Geriatric medicine in general, and geriatric rehabilitation in particular, is increasingly recognizing the importance of the emerging concept of "Intrinsic Capacity". Attempting to operationalize and deepen the understanding of this novel entity, contemporary aging theorists consistently return to the geriatric core issues of locomotion, neuromuscular function, sensorium (hearing/vision), and physical vitality (frailty/resilience/homeostasis/reserve), alongside cognitive, psychological, and social function [12–14].

The concepts of frailty and resilience among older people are useful tools to help understand and quantify the increasing heterogeneity across multiple biological systems, which typifies the aging process [15–17]. This observation is true for not only trajectories of disease, function and survival, but also for rehabilitation potential. The gap between the concepts of biological and chronological aging among the oldest old emphasizes the importance of accurate personalized assessment, covering the wide range of geriatric, functional, and rehabilitation potential and goals [18, 19]. In the absence of such individualized and multi-dimensional assessment, critical decisions throughout the patients' care concerning ICU admission, continuation of intensive care, as well as post-ICU placement are likely to be based erroneously upon chronological age alone [20, 21].

## Multidisciplinary approach

**Fig. 2:**
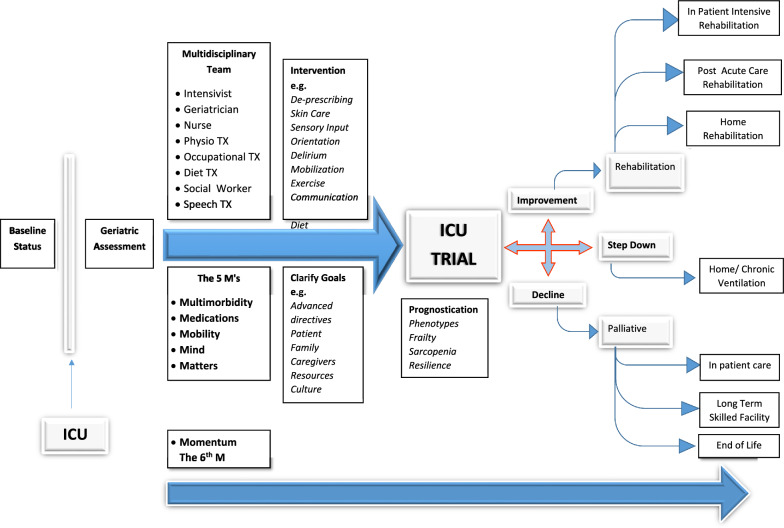
The trajectory of critically ill very old patients through ICU admission and post discharge. The trajectory of very old critically ill patients includes baseline comorbidity and function, ICU multidisciplinary assessment and treatment, time limited trials and defining goals of care, rehabilitation assessment and planning. Post ICU trajectories include short and long term rehabilitation in acute and subsequently chronic/home care as well as end of life decision making

A multidisciplinary team embedded in the ICU is a prerequisite for optimal geriatric rehabilitation of the very old ICU patient. These include physicians (intensivists, geriatricians, and rehabilitation specialists, as well as endocrinologist and palliative care specialists as needed), nurses, physiotherapists, occupational therapists, dieticians, social workers and speech therapists (Fig. 2). Regular assessment as well as ongoing follow-up are essential to accurately determine the short and long-term rehabilitation potential in view of the patient's chronic conditions, acute illness, medications, physical and mental reserve and treatment preferences. Prognostication in this patient population is challenging due to multi-morbidity, complexity and frailty, however identification of different phenotypes has been shown to assist in decision making and in tailoring interventions including early rehabilitation [22]. Multidisciplinary discussions involving the ICU team, members of the rehabilitation team, patients and family members are required to facilitate shared decision-making concerning short-term rehabilitation goals [8], and if necessary, the replacement of invasive and/or intensive treatments by palliative oriented care [23, 24].

## Geriatric assessment and goal setting in the ICU

Primary goals of care in the treatment of very old patients in intensive care include organ support and recovery of organ function, improved survival rates and prevention of hospital acquired complications. From a geriatric medicine perspective, goals of care also include improved physical and cognitive function, maintenance of a reasonable quality of life and provision of adequate rehabilitation during and following hospital admission. In the geriatric patient population, comprehensive geriatric assessment (CGA) is the standard tool to identify risks for adverse outcomes and prioritise treatment targets in the long term, such as restoration of mobility [25]. Although evidence for the beneficial role of CGA in the ICU is still missing [26], it is known that patients receiving a CGA on hospital admission are more likely to survive and be discharged to their own homes [27]. Even though it appears unrealistic to have a complete CGA in the ICU due to the incapacity of the patient and resource constraints, abbreviated versions of the CGA can be employed to extract pivotal information. For example, the 5Ms framework focuses on core elements of the CGA: 'Mobility', 'Mind', 'Medications', 'Multi-complexity', and what 'Matters most' for older patients and has been recommended for geriatric emergency care [28–30]. The feasibility of conducting core elements of the CGA within the acute care setting is challenging and remains an area to be further studied in order to facilitate implementation. Among the limited research that does exist, evidence supports common tools for the assessment of frailty and cognition [14, 31, 32] (Table 1).

**Table 1:** Title core elements for geriatric assessment in and following ICU: the 6M's

Core domain	Site	Agent of care	Timing	Example of intervention
*Mobility*				
Mobilization Passive/active Posturing	ICU/ Step down	ICU Nursing staff/ Physiotherapist	Post admission Stable patient	Positioning in & out of bed; Passive range of movements
			Post admission Stable patient /awake/ cooperative	Active Range of movements/Transfers/ Strength exercise/Balance/ Dynamic/static/coordination
*Mind*				
Cognitive assessment Delirium Assessment	ICU	ICU Nursing staff	Post admission/ Awake/ or RASS > -2	Delirium (hypo / hyperactive) CAM
	Step down	GNP	Awake	CGA
Cognition/orientation	ICU	ICU Nursing staff	Post admission Communicating	Follow up
	Step down	GNP		CGA
Psychological Status/mood/sleep	ICU	ICU Nursing staff	Post admission/ Communicating/ No delirium	Follow up
	Step down	GNP		CGA
Competency/capacity	ICU	GNP/ Geriatrician/ Psychiatrist	Post admission/ Communicating/ No delirium/ No narcotics/ sedatives	Kitchen Picture Test [39]
	Step down/ General ward			
Communication	ICU	Speech Therapist/ Nursing staff	Post admission/ Communicating/ No delirium/ No narcotics/ sedatives	
	Step down/ General ward			
Sensory Integrity/Assistive sensory aids	ICU	Ophthalmologist Otolaryngologist	Post admission	Family and patient interview/Past Medical History/Physical assessment/ ENT/ophthalmologist
	Step down/ General Ward			
Pain/nausea	ICU	ICU Nursing staff/ Pain clinic consultants/ GNP for delirious patients	From day one of admission	BPS for sedated patient/ VAS for alert patient
	Step down/ General ward			
*Medications*				
Polypharmacy	ICU	Physician/GNP Clinical Pharmacist	Continuous	Assess chronic medications/withholding/reducing/ restarting according to patient's condition
	Step down General Ward		Continuous	Adjusting medications to patient's condition Preparation for stable dosing and long-term treatment
De-prescribing: Psychoactive/Central acting Drugs	ICU	Physician/ GNP	From admission, According to patient's condition. Aim for Minimum dose	BEERS [40] STOPP START [41]
	Step down/ General Ward			
*Multimorbidity*				
Sphincter Control/ Autonomic function	ICU/ Step down/ General Ward	Nursing staff follow up	From admission, according to patient's condition	
Skin care/pressure sores	ICU/ Step down/ General Ward	Nursing staff follow up	Continuous assessment from admission, according to patient's condition	NORTON scale for risk assessment/ Daily skin assessment [42]
Oral care	ICU/ Step down/ General Ward	ICU Nursing staff	Daily assessment and standard care	
Swallowing assessment	ICU/ Step down/ General Ward	Condition dependent Not intubated Awake and cooperative Routing feeding-Nursing Staff	Continuous assessment from admission, according to patient's condition	Staff report swallowing difficulties/ known pathology/ Speech Therapist/ ENT
Nutritional Status	ICU/ Step Down/ General Ward	Dietician	Continuous assessment from admission, according to patient's condition	
Frailty/Physiological reserve	ICU	Nursing staff	Assessed on admission	History/Screening tools. CFS [43]
	Step Down/General Ward	Physician/ GNP	Assessed on admission	Diagnostic tools e.g. HANDGRIP/ TUG
*What Matters Most*				
Preferences/goals/ cultural background/Integrity/dignity	ICU/ Step down/ General Ward	Physician/Nursing/ GNP/ Palliative care consultation/ Social Worker (S/W)/ Advanced directives/ Next of Kin (NOK)/ Surrogates	Conscious and competent patients-Assessed on admission or earliest timing possible	Patient preferences/advance directives/surrogates/custodians/family interview
*Momentum*				
Prognostics/Trajectory of critical illness	ICU/ Step Down/ General Ward	Physicians	Updating according to available data	Time-limited trial with short term goals/ monitor biomarkers
Motivation/Compliance/Resilience	Step Down/General Ward	Physician/Nursing/GNP/ Physiotherapists/Social workers/ all staff	Conscious/Competent patient at earliest timing possible	
Social support/Family support	ICU/ Step Down/General Ward	S/W	Continuous assessment from admission, background, acute and chronic conditions changes in patient's condition	Family and other caregivers/Barriers to future care/ Finances

BEERS—Beers Criteria for Potentially Inappropriate Medication, BPS—Behavioural Pain Scale, CAM—Confusion Assessment Method, CFS—Clinical Frailty Scale, CGA—Complete Geriatric Assessment, ENT—Ear Nose and Throat, GNP—Geriatric Nurse Practitioner, ICU—Intensive Care Unit, NOK—Next of Kin, RASS—Richmond Agitation and Sedation Scale, STOPP-START—Screening Tool of Older People's Prescriptions (STOPP), Screening Tool to Alert to Right Treatment (START), S/W—Social Worker, TUG—Timed Up and Go, VAS—Visual Analog Scale

Rather than a single static geriatric assessment, it is often the older patient's trajectory over time in the ICU that is of critical importance in both prognostication and decision-making [33, 34] (Fig. 2). The patient's changing state is a reflection of their resilience, i.e. their ability to "bounce back". The overall direction (Momentum) of older patients' response over time, reflects not only their response to disease/organ-specific treatment, but is also highly influenced by the core geriatric concepts of frailty/intrinsic capacity/ resilience. Indeed, taken as the sum of these core determinants within the critical care scenario, we would suggest that an assessment of the patient's "Momentum" over time might be seen as the 6th "M" to be considered in addition to the 5Ms framework [35].

The findings of the geriatric assessment have to be integrated with information about the critical illness in order to determine goals and potential care trajectories [22]. In that context, the focus on patient-centered outcome measures means [35]:

- to include the patient's individual preferences and beliefs in order to frame choices within the dimensions of benefit as well as harms and burdens of care,
- to favour therapies that optimize benefit with regard to quality of life and minimize harm,
- to consider feasibility of interventions in the patient's and caregivers' social and cultural context.

The effective and sustainable implementation of the above processes requires a designated coordinator with a medical or advanced nursing background [36].

Early assessment of the potential involvement of significant family members and caregivers is an important prognostic factor for functional outcome [37], and an integral step towards facilitating their participation in aspects of both critical care as well as rehabilitation. An assessment of social support and sensitivity towards cultural background and core values is essential in order to help promote and optimize both patient and family compliance with rehabilitation goals and ensure meaningful communication [38].

## Assessment of rehabilitation potential

The overall goal of the initial assessment is to aid in prognosticating the functional outcome of intensive care in very old individuals. Existing literature gathered over the last two decades emphasizes the value of assessment of frailty, sarcopenia, functional and cognitive status, and functional performance measures, in addition to illness severity at the time of ICU admission [14, 31, 32]. Predictions of rehabilitation potential and outcomes are difficult, and very few evidence-based recommendations to operationalize this concept exist. However, among the existing literature, consistent associations are noted between multi-morbidity, functional status, and particularly frailty as predictors of both short and long-term function among ICU older patients [9, 31, 44–47]. Fuest et al. described an intelligent-based algorithm for mobilization protocols in four clusters of patients, aimed at increasing the likelihood of discharging patients to their home [48]. They found that most patients, including the cluster of frail patients and non-frail old patients, benefited from frequent mobilization efforts.

Although screening for delirium as an obstacle for rehabilitation has become an essential component of intensive care, other components of the neurocognitive status, including pre-existing cognitive impairments, as well as brief screening for decision-making capacity and competence, require close attention. The close relationship between pain, agitation, and delirium with generalized neurocognitive status has been recognized in the PAD (Pain, Agitation and Delirium) guidelines [49, 50], which were subsequently updated in 2018 to also include sleep and immobility in the PADIS (Pain, Agitation, Delirium, Immobility, Sleep) guidelines [50, 51]. The importance of family and caregiver involvement is also recognized as a positive prognostic factor [37], and is included in the ABCDEF bundle (Assess-manage pain; Breathing trials; Choice of analgesic and sedation; Delirium; Early mobilization and Exercise; Family engagement and empowerment)- all of which have been found to be associated with good functional outcomes during and post-ICU [52].

Other rate-limiting factors to the rehabilitation process include cardiovascular and pulmonary reserve, in conjunction with severe sarcopenia, ongoing infection, catabolic, and inflammatory status, which may worsen sarcopenia, further complicating the potential for rehabilitation. Functional assessment of performance measures should be performed to aid in assessment of potential: gait, balance, coordination, strength, range of movement, as well as locomotion; assessment of sensory integrity, autonomic and involuntary function, bowel and sphincter control; skin integrity, nutritional status, swallowing and feeding concerns; as well as communication and assistive aids. Unnecessary lines and catheters should be removed as soon as possible. The question arises concerning when is the optimal timing for assessment of more complex motor, sensory and autonomic function. Clearly clinical judgement is necessary, accounting for the acute nature of the patient's condition. Nonetheless, once the patient is stable, early assessment rather than later is likely to lead to earlier intervention, which in turn is likely to prevent further subsequent deterioration of function. According to the patient's status, the more advanced functional measures might be reserved for assessment prior to discharge to step-down post ICU care.

For convenience we have listed several common assessment tools. These were chosen since they are all very common, standardized, well validated clinical scales, widely used in everyday clinical practice, and accepted in current up to date literature (Table 1 and Supplement Table 1).

## Rehabilitation in the ICU

Despite methodological difficulties, lack of standardized treatment protocols, lack of age-dependent stratification, differing criteria for inclusion/exclusion, as well as a generalized under-representation of very old patients in the relevant studies, most studies tend to confirm the proven benefits seen from early mobilization and active exercise programs, whilst confirming a high degree of safety [50, 51, 53, 54].

Physiotherapy aims at the restoration and improvement of neuromuscular integrity and function, strength, coordination, locomotion, and mobilization. Accepted physiotherapy techniques aimed at ICU patients tend to be inclusive of all ages, limited by the patients’ level of performance alone. These include positioning, mobilization, manual hyperinflation, percussion, vibrations, suction, cough, and breathing exercises [55]. Nonetheless, it is likely that age-associated premorbid conditions (e.g. musculoskeletal degenerative disease, chronic pain, sarcopenia) are likely to complicate rehabilitation, and further emphasize the need for individualized specific care for the very old ICU patient [56, 57].

Occupational therapy aims to optimize an individual's "occupation" in meaningful activities, aiming to develop, improve or regain mental and physical performance [58, 59]. Occupational therapy in the ICU setting when relevant, aims at maximizing optimal sensory input, assistive technology, cognitive treatment, self-care skills, as well as specific postural aids and splints. Closely coordinated work between dietitians and speech therapists aiming to overcome swallowing difficulty as well as to optimize dietary intake. The growing availability of virtual reality within the rehabilitation scenario is finding its way into the ICU, both for cognitive as well as physical exercise training. Additionally, its use is being examined as a potential preventive tool for delirium, using its potential to induce relaxation and counteract the noxious cognitive and sensory stimuli of the ICU environment [50, 51, 60, 61]. As awareness grows concerning the potential usefulness of early intervention of both existing and novel rehabilitation modalities, further exploration of the feasibility and implementation challenges of these interventions in the ICU may be warranted.

The critically important role of specialized and skilled nursing care is often the final common pathway in patient care, integrating the results of all the multidisciplinary treatment modalities into the day-by-day care. Translating short-term goals of the specific rehabilitation modalities into everyday practice requires mindful nursing care which is actively oriented towards the rehabilitation goals. Thus, for example the primary goals nursing care of the very old ICU patient may be aimed at optimal skin care, pain control, bowel habit, oral hygiene, early mobilization, attention to sensory input (eyeglasses and hearing aids), improved communication and orientation, reduced restraints and environmental stressors, and improved family involvement and support. However, such steps are likely to induce secondary effects and benefits such as reduced delirium and psychomotor agitation, improved sleep and mood disorders, reduction in stress and levels of depression, as well as improved disposition towards their environment and greater compliance with rehabilitation interventions [62, 63]. Successful rehabilitation frequently requires optimal control of disturbing symptoms, and a palliative assessment may be indicated. Furthermore, it may become apparent during the ICU admission that rather than rehabilitation and recovery, the primary goals of care are oriented to the end of life. In such cases the involvement and support by palliation teams may improve patient and family stress and suffering and allow for an easier transition through the ICU stay towards end-of-life care [64, 65]. (Table 1 and Supplement Table 1).

## Rehabilitation interventions after discharge from the ICU

The common sequelae of critical illness among the very old survivors of ICU with severe generalized deconditioning and functional decline are described in Table 2, and may be collectively termed "Post Intensive Care Syndrome"—PICS [37, 66–68]. PICS is a complex syndrome of multiple physiological cognitive and psychological impairments following intensive care treatment. Examples include intensive care acquired weakness, dysfunctional swallowing, memory loss, delirium, post-traumatic stress disorder and depression [68]. These long-term effects may also influence family members (PICS-F) who have to cope with these functional changes and necessitate a family-centered rehabilitation approach [69].

**Table 2:** Common sequelae following ICU admission among very old people

Deconditioning and weakness	Cognitive impairment
ICU–acquired weakness Neuropathy Myopathy Sarcopenia Frailty Medication induced	Dysfunction across multiple domains Impaired decisional capacity/competence
	Psychological disorders
	Confusion Anxiety Depression Post-traumatic stress Psychosis
Feeding and Nutritional Problems	Behavioural
Oral/dental problems Swallowing disorder Dysphagia Post-intubation damage Reduced intake Anorexia/cachexia Malabsorption Catabolic state	Psychomotor agitation Sleep disorder Negative disposition/reduced compliance Reduced interaction with environment Withdrawal
	Sensory impairment
	Hearing Vision Taste Smell
Skin and Wounds	
Breakdown Infections Pressure Sores Delayed Healing	
	Inflammatory status
	Catabolic state Inflammation Ongoing infections Immunocompromised/suppressed
Reduced Physiological Reserve	
Cardiovascular Hemodynamic Pulmonary Endocrine homeostasis Renal Immunological Bone metabolism	
	Functional Decline
	Immobility Incontinence Dependence in Basic Activities/Function
Pain	Procedure related morbidity
Musculoskeletal system Contractures/Range of movement Prolonged immobility Invasive procedures	IV lines Catheters Drains
Delirium	
Associated with previous impairment	
Predicts subsequent impairment	

Despite the common preconception of poor long-term rehabilitation potential and outcomes among very old ICU survivors, little high-quality evidence-based research actually exists to either support or disclaim this view. Findings that do exist are often inconclusive, and conflicting. Thus, for example, a Canadian study of ICU patients aged 80+ showed that 25% of subjects had survived and returned to their baseline function after 12 months [32]. These findings stand in contrast to a Finnish study of people age 80+, which showed that among the 62% surviving to 12 months, 78% actually reached their baseline function [31]. Indeed current NICE clinical guidelines for rehabilitation following critical illness make no distinction based upon chronological age alone [70, 71]. The negative effects of ICU admission on close family members and informal caregivers is also recognized, both short and long-term [72]. These in turn may result in additional subsequent negative repercussions upon the patient’s rehabilitation potential.

As important as initial triage, the “seamless transition of care” at the time of discharge from ICU is a critical step in the patient’s trajectory (Fig. 2). As emphasized by UK guidelines, the assessment of subsequent rehabilitation potential at the time of discharge, is a vital determinant of the subsequent degree of appropriate care, occurring at the critical moment of “stepping-down” [71]. While multidisciplinary assessment at ICU admission may be both ambitious and challenging, it becomes gradually more realistic and feasible to perform increasingly complex core elements of assessment along the trajectory through ICU, as the patient stabilizes and their rehabilitation potential becomes apparent. Thus, the CGA might be perceived as an evolving assessment, culminating in a truly comprehensive picture prior to decision-making at the moment of ICU discharge and step-down. Often reflecting the patient's degree of existing resilience, cognition, neuromuscular and cardiovascular reserves, as well as motivation, compliance and disposition towards rehabilitation, the intensity of different geriatric rehabilitation settings are varied [73, 74]. In general, step down from ICU to general medical wards within the general hospital precedes subsequent transfer to rehabilitation facilities. The UK guidelines remain relevant for these intermediate settings. Nonetheless, there is a major need to encourage the widespread implementation of such guidelines, emphasizing the rehabilitation needs of the patient at the time of transfer out of the ICU so that the accepting ward continues the relevant assessment and planning.

Rehabilitation may be provided in different modalities and intensity depending on the patients' potential and capacity. Variously named as geriatric rehabilitation, sub-acute care, post-acute care, and transitional care, there is often a very large variability in definition, standards and intensity of care, and costs (Fig. 2). In the absence of high-quality research, it is largely unclear how different care-settings actually affect patient outcomes. Amongst the oldest old geriatric rehabilitation patient, the entire spectrum of geriatric medicine comes into the forefront. The range of different services among different healthcare systems reflect the ongoing debate concerning the optimal rehabilitation setting for these complex and challenging patients [67].

## Home rehabilitation

In addition to intense geriatric rehabilitation, the area of home rehabilitation is becoming increasingly popular. Indeed, home care in general is an area of rapid growth, having received renewed interest following the recent COVID-19 pandemic [75]. The recent growth of innovative technology, enabling for example smart homes, remote monitoring, telemedicine, as well as enormous financial incentives to reduce the burden on in-patient hospital beds: all these together have led to renewed interest and a surge in the provision of home rehabilitation [76]. Whilst contingent upon a high degree of both patient, family, and caregiver compliance, home care is commonly a preferred option by the dyad of patient and family caregivers and seen as a financial win–win for the Healthcare provider in many places.

Like many aspects of rehabilitation among the oldest old ICU patient, there still is very little evidence-based research concerning home rehabilitation. Among existing research, the direction of findings is generally supportive of the positive home rehabilitation outcomes among survivors of ICU, across measures of locomotion, quality of life, safety, respiratory function as well as financial viability of home based care [76, 77]. Furthermore, among patients returning home following ICU despite failure to wean from invasive ventilation, in some countries home hospital is viewed as a preferred option among many prolonged mechanically ventilated patients, among whom quality of life, mood, and measures of caregiver stress consistently support home versus long-term care facilities [78–80].

## Common barriers

The common barriers to rehabilitation among ICU patients in and following ICU admission, are more frequent among older people (Table 3). Patient-centered barriers include: fatigue/weakness/pain/polypharmacy/anxiety/poor motivation/confusion/restraints. Common recurring themes to emerge among ICU patients across all ages, emphasize loss of self-autonomy and competence, dehumanization and a need for recalibration of self-identity [81]. It seems likely that these symptoms are more common or pronounced among the very old patients, resonating with pre-existing themes shared in common with aging. It is essential to strive towards early identification of potential barriers which may complicate subsequent discharge and site of care, and to identify potential surrogate decision-makers in the absence of advanced planning directives.

**Table 3:** Potential barriers to rehabilitation for the very old critically ill patients

Patients Centered	Environmental
Fatigue	Inadequate availability of rehabilitation therapists
Weakness	Inadequate availability of rehabilitation equipment
Pain	Negative perception of rehabilitation by staff members
Polypharmacy	Inequalities in provision of rehabilitation for very old
Anxiety/depression	
Confusion	Organizational
Agitation	Poor evidence base for this patient population
Ongoing delirium	Financial constraints
Pressure sores	Attitudes of Stakeholders and Policy makers
Lack of Motivation	Local and national health care policy
Poor compliance	Ethical and cultural norms
Need for restraints	Ageism
Family and Caregiver centered	
Inadequate social support	
Inadequate family support	
Caregiver burden and burnout	
Lack of consensus concerning goals	
Financial constraints	

In addition to patient-centered factors, environmental factors influencing rehabilitation outcomes are numerous [82]. The quantitative lack of resources include low staffing-levels of multidisciplinary healthcare professionals specializing in very old patients; low-frequency or complete lack of geriatric rehabilitation multidisciplinary meetings; lack of specialized rehabilitation equipment; as well as poor availability of subsequent geriatric rehabilitation beds for older people, in and out of acute care. Community based facilities for the rehabilitation of the very old, as well as an adequate infrastructure of knowledgeable, multi-disciplinary teams for home rehabilitation and support are lacking. Thus, there is a need for specialized and mindful planning of potential rehabilitation services and care plans in the community, as well as education programs for the relevant stakeholders, health-care professionals, patients, family members and other caregivers.

No less important are the qualitative barriers to geriatric rehabilitation, especially the lack of acknowledgment and under-appreciation of the necessity and benefits of early geriatric rehabilitation among older critically ill patients. This under appreciation of the benefits, or disinclination to recognize the importance of rehabilitation for the oldest old patient is ultimately responsible for the inadequate delivery of appropriate geriatric rehabilitation, leading to a self-fulfilling prophecy [83].

Inequalities in the provision of geriatric rehabilitation services is perhaps one of the most obstinate barriers to be faced. Limited resources, local and national health policy, stakeholders, as well as financial incentives are but some of the complex factors to be confronted in order to address this pressing issue [71]. Educational steps to revert this imbalance should be aimed not only at ground-level health professionals, but perhaps more importantly, at health-policy and decision-makers. Thus, a multi-tiered approach is necessary to address the current state of affairs whereby the geriatric rehabilitation needs of critically ill old people remain largely unmet [84]. In order to make a more efficient change, further exploration of strategies to both identify and mitigate these numerous barriers may be warranted.

## Conclusions

Very old critically ill patients are a rapidly growing population in intensive care units, posing a great challenge in acute care and rehabilitation, due to multi-morbidity, disease complexity and frailty. In parallel to and following treatment for the acute illness, careful geriatric assessment may help evaluate rehabilitation potential, taking into consideration patient heterogeneity in terms of functional capacity, frailty, and resilience. Multi-disciplinary assessment is required for planning optimal rehabilitation, cognitive and sensory function and early mobilization, during and following the ICU stay. A comprehensive multi-system checklist may guide healthcare workers in assessment and planning. Measures to prevent long term sequela of critical illness and to overcome barriers to rehabilitation should be implemented. Family support, not only during but also following ICU care is essential for the continuum of rehabilitation post admission and the provision of good functional outcomes.

### Supplementary Information

Supplementary material 1.

## Data Availability

N/A.
