# Supplementary material for: Critical care beyond organ support: the importance of geriatric rehabilitation
Source: Ann Intensive Care. 2024 May 10;14:71. doi: 10.1186/s13613-024-01306-1 (PMC11087448; doi:10.1186/s13613-024-01306-1)
Supplement: Supplementary file 1 — Supplementary material 1. [file 13613_2024_1306_MOESM1_ESM.docx]

**Supplement Table 1. Checklist for the Assessment and Management of Geriatric Patients in Acute Care**

|  | **Intervention** | **Education** |
| --- | --- | --- |
| **Identifying patients at risk for delirium** | **Risk** **assessment**.  All ICU patients are at risk for delirium.   - Patients > 75 y.o - Patients > 65 y.o with:   - Cognitive impairment   - Functional impairment   - Risk of sleep deprivation   - Risk of decreased mobility/ immobility   - Visual/ hearing impairment with or without need for aids.   - Polypharmacy   - Medications associated with a risk of delirium | **Staff education,**  **Increased awareness among healthcare professionals**   - Delirium prevention guidelines |
| **Delirium prevention** | **Intervention:**   - ICU+ WARD   - Early removal of catheters and lines   - Minimizing physical restriction   - Maintaining orientation   - Physiotherapy, Early mobilization   - Presence of family members or friends   - Use of hearing and visual aids   - Maintaining sleep hygiene   - Minimizing use of medications associated with risk of delirium   - Minimizing use of benzodiazepines | **Staff education**   - Delirium prevention guidelines   **Patient and family education**   - Continuous orientation for the patient - Family members or friend's visits   - Assistance with feeding   - Encourage mobility - Use of hearing and vision aids - Sleep hygiene education |
| **Early detection delirium** | **Intervention:**   - ICU   - CAM ICU - WARD   - CAM     - On admission     - Daily assessment   - Delirium treatment protocol initiation | **Staff education**   - CAM - Delirium treatment guidelines |
| **Cognition** | **Cognitive assessment**   - ICU & WARD - Background - Functional and cognitive baseline - Evaluation and prognosis - Current assessment of cognition - Cooperation - Motivation - Acute delirium events | **Staff education**   - Basic Cognitive assessment - Background Cognitive status - Recording acute delirium events |
| **Pain management** | - ICU - Continuous pain assessment (BPS) in sedated patients - Adjustment/ weaning pain treatment - Balancing analgesia vs sedation - Alternatives to continuous anesthesia - WARD   - Pain management protocol for the elderly   - Continuous pain assessment   - Adjustment/ weaning of pain treatment   - Non opioid orientation   - Multimodal analgesia   - Proactive consultation of pain clinic | **Staff education**   - Pain assessment - Atypical manifestations - Misconceptions about opioid tolerance and addiction. - Treatment guidelines in the geriatric population - BEERS CRITERIA/STOP-START |
| **Sedation** | - ICU - Sedation level assessment - RASS scale depth 0 to -1 - Daily Wake Up Test - Adjustment/ weaning orientation - BEERS CRITERIA/STOP-START - Prevention of physical/chemical restraint - Patient's family presence at the bedside | **Staff education**   - Sedation level assessment - Treatment guidelines in the geriatric population- BEERS CRITERIA/STOP-START - Prevention of physical/chemical restraint     **Patient and family education** |
| **Depression** |  |  |
|  | - ICU+ WARD - Patient Health Questionnaire -2 (PHQ-2) - Weekly Screening | **Staff education**   - Patient Health Questionnaire -2 (PHQ-2) |
|  | **Intervention:**   - Nonpharmacological: - Encouraging mobility - Exposure to sunlight - Moderate physical activity - Patient and family education | **Patient and family education**   - Visiting - Encouraging mobility - Moderate physical activity |
| **Sleep hygiene** | **Sleep hygiene assessment** |  |
|  | **Intervention**  **ICU**   - - Reduction of loud noises and alarms   - Dimming lights at night   - Adjustment of medication administration & treatment times   **WARD**   - - Reducing sleep during the day   - Exposure to sunlight during the day hours | **Staff education**   - Reduction of loud noises - Dimming lights at night - Adjusting of treatment administration times - **Patient and family education** - Reducing sleep during the day - Exposure to sunlight during the day hours |
| **Comprehensive Geriatric Assessment (CGA)** | **WARD**   - - Early after hospitalization   - Critical review of drug therapy   - Adjusting medications to the   patient's changing condition   - - Weaning from sedative   - painkillers |  |
| **Frailty** | - - e.g. Clinical Frailty Scale (CFS) |  |
| **Respiratory Management** | **ICU**   - Respiratory **a**ssessment - Optimal ventilation and oxygenation - Inhalations - Cough devices - Reduced sedation / daily sedation vacation - Respiratory weaning - Physiotherapy - Muscle strength assessment - Mobilization in bed/ Out of Bed - Pain assessment - Early tracheostomy | **Staff education**   - Protective ventilation - Weaning strategies - Oxygen treatment guidelines - Physiotherapy   **Patient and family education**   - Physiotherapy - Respiratory self-training |
| **Mobility** | - Prioritization for physiotherapy - frailty - risk of delirium/ delirium   • Pre- habilitation   - Medical mobility restrictions - Mobility duration   - single long sessions   - multiple sessions - Active /passive activation - Respiratory therapy - Mobility in bed, sitting position. - OOB   - Full weight bearing   - Partial weight bearing / lever - Mobility assistance   - Walker, cane   - Boot, walking cast. | **Staff education**   - Functional background - Current level of functioning - Level of assistance for transitions. - Adjusting pain medication for functioning. - Assessing cooperation   **Patient and family education**   - Mobilization support and training - Pre- habilitation planning and support - Mobility assistance devices |
| **Nutrition** | ICU + WARD  **Nutrition**   - Assessment and optimization of patient's nutritional status | **Staff and family education**   - Nutrition protocols, supplements, targets |
| **Social** | - ICU+ WARD - Social status - Advance directives - Legal guardian appointment - Shared decision making - Community support planning - Community / home rehabilitation planning | **Patient and family education**   - Involvement of the patient's family - Shared decision making - Legal guardian appointment - Advance directives and patient preferences - Social support, residence - Preference of rehabilitation - Home care and rehabilitation - Management of caregiver burden |
